# Long-Lasting Cortical Reorganization as the Result of Motor Imagery of Throwing a Ball in a Virtual Tennis Court

**DOI:** 10.3389/fpsyg.2015.01869

**Published:** 2015-12-01

**Authors:** Ana M. Cebolla, Mathieu Petieau, Carlos Cevallos, Axelle Leroy, Bernard Dan, Guy Cheron

**Affiliations:** ^1^Laboratory of Neurophysiology and Movement Biomechanics, ULB Neuroscience Institute, Université Libre de Bruxelles, Brussels, Belgium; ^2^Department of Neurology, Hopital Universitaire des Enfants Reine Fabiola, Université Libre de Bruxelles, Brussels, Belgium; ^3^Haute École Condorcet, Charleroi, Belgium; ^4^Laboratory of Electrophysiology, Université de Mons-Hainaut, Mons, Belgium

**Keywords:** motor imagery, EEG, virtual reality, N300, ERSP, ITC, beta, ERD

## Abstract

In order to characterize the neural signature of a motor imagery (MI) task, the present study investigates for the first time the oscillation characteristics including both of the time-frequency measurements, event related spectral perturbation and intertrial coherence (ITC) underlying the variations in the temporal measurements (event related potentials, ERP) directly related to a MI task. We hypothesize that significant variations in both of the time-frequency measurements underlie the specific changes in the ERP directly related to MI. For the MI task, we chose a simple everyday task (throwing a tennis ball), that does not require any particular motor expertise, set within the controlled virtual reality scenario of a tennis court. When compared to the rest condition a consistent, long-lasting negative fronto-central ERP wave was accompanied by significant changes in both time frequency measurements suggesting long-lasting cortical activity reorganization. The ERP wave was characterized by two peaks at about 300 ms (N300) and 1000 ms (N1000). The N300 component was centrally localized on the scalp and was accompanied by significant phase consistency in the delta brain rhythms in the contralateral central scalp areas. The N1000 component spread wider centrally and was accompanied by a significant power decrease (or event related desynchronization) in low beta brain rhythms localized in fronto-precentral and parieto-occipital scalp areas and also by a significant power increase (or event related synchronization) in theta brain rhythms spreading fronto-centrally. During the transition from N300 to N1000, a contralateral alpha (mu) as well as post-central and parieto-theta rhythms occurred. The visual representation of movement formed in the minds of participants might underlie a top-down process from the fronto-central areas which is reflected by the amplitude changes observed in the fronto-central ERPs and by the significant phase synchrony in contralateral fronto-central delta and contralateral central mu to parietal theta presented here.

## Introduction

Motor imagery (MI) is the mental simulation of a given action (Jeannerod, 1994; Jeannerod and Decety, 1995). MI is free from interference by real movement, which makes it interesting when investigating the neural organization of motor control. To this end, MI coupled with the event related potentials (ERP) technique has been widely used in both healthy (Cheron and Borenstein, 1992; Abbruzzese et al., 1999; Rossini et al., 1999) and clinical populations (Filippi et al., 2001; Volz et al., 2015). As a practical application, MI is used as a therapy to facilitate motor recovery after spinal cord injury (Cramer et al., 2007; Grangeon et al., 2012) or stroke (Dijkerman et al., 2004; Zich et al., 2015) and is also employed as a training technique for improving motor performance in sports (Wang et al., 2014; Di Rienzo et al., 2015; Ridderinkhof and Brass, 2015).

The brain structures participating in MI have been exhaustively depicted by means of fMRI, which reveal the involvement of the primary motor cortex, premotor cortex, supplementary motor cortex, cingulate cortex, parietal cortex, basal ganglia, and cerebellum (Beisteiner et al., 1995; Decety, 1996; Grafton et al., 1996; Porro et al., 1996; Sirigu et al., 1996; Deiber et al., 1998; Lotze et al., 1999; Gerardin et al., 2000; Naito et al., 2002; Hanakawa et al., 2003; Dechent et al., 2004; Meister et al., 2004; Stinear et al., 2006; Guillot et al., 2008; Jiang et al., 2015; Krippl et al., 2015). It is well known that the electroencephalogram (EEG) provides excellent temporal resolution with respect to fMRI. On top, while fMRI is based on the relation between neuronal activation and regional blood flow, the EEG technique has a more direct access to the brain’s electrical activity, one of the essential mechanisms of neuronal communication. Ongoing brain rhythms represent “universal codes,” from which information transfer is established in the brain (Friston et al., 1997; Singer, 1999). Thus the time-frequency characteristics of EEG oscillations, notably power spectrum and phase synchrony, may elucidate mechanisms of brain function such as MI, and thus complement anatomical information from fMRI or even from advanced inverse solution models (Cebolla et al., 2014).

The temporal domain measurement of ongoing oscillations (that is ERP) has been reliably applied to MI (Cunnington et al., 1996; Yahagi and Kasai, 1999; Romero et al., 2000; Bastos et al., 2011; Machado et al., 2013; Tabrizi et al., 2013). The time-frequency domain measurements of ongoing oscillations (power spectrum and phase consistency) related to MI made possible operative brain computing interfaces (Neuper et al., 2005; Pfurtscheller et al., 2013; Zhang et al., 2015). In most of cases, the power spectrum and phase synchrony characteristics that underlie MI represent the cerebral output carrying the movement program toward the interface that, in turn, links brain to an external device or machine which will then execute the movement or function (Neuper et al., 2005; Pfurtscheller et al., 2013; Zhang et al., 2015). To a lesser extent, hybrid brain computing interfaces combine the ongoing brain oscillatory characteristics directly associated to the MI with a concomitant, but “non-directly MI-related” visual evoked potential (VEP). The frequency of stimulation of this specific type (steady state-) of VEP differs greatly from the day-to-day stimulation (Pfurtscheller et al., 2010). Recent studies of power spectrum variations in MI have shown a related alpha-mu and beta power decrease (Thomas et al., 2012; Zhou and Wan, 2012; Chen et al., 2013; Friedrich et al., 2013; Hashimoto and Ushiba, 2013; Llanos et al., 2013; Yi et al., 2013; Brinkman et al., 2014; Osuagwu and Vuckovic, 2014; Vucinovic et al., 2014).

In order to characterize the neural signature of a MI task, the present study investigates its oscillation characteristics, including both of the time-frequency measurements [event related spectral perturbation (ERSP) and intertrial coherence (ITC; Delorme and Makeig, 2004)], which underlie the specific variations in temporal measurements (ERP) of a MI task. We hypothesize that significant variations in both time-frequency measurements underlie the specific changes in the ERP directly related to MI. To our knowledge, this is the first study to include both of the time-frequency measurements that underlie a related MI ERP. For the MI task, we chose a simple, everyday action that does not require any particular motor skill:, throwing a tennis ball with the dominant upper limb in a standing position, within the controlled virtual reality scenario of a tennis court. In this way the task studied here could be used as an ecological movement reference for specific throwing movements in sports that are normally executed in a standing position, such as the service in tennis.

## Materials and Methods

### Participants and Conditions

The data were collected from 11 right-handed (96.3% ± 8.1) by means of the Handedness inventory (Oldfield, 1971), healthy volunteers (five females and six males, mean age: 20.5 ± 3.3 years). These participants had previously been qualified as good imagers (5.95 ± 0.95) score determined by means of the French version of the Movement Imagery Questionnaire-Revised (Hall and Martin, 1997; Butler et al., 2012). They were all in good health and free from neurological disease. The procedures were approved by the local ethics committee of the university and conformed to the Declaration of Helsinki.

The EEG was recorded with 128 channels (ANT neuro system) at a sampling frequency of 2048 Hz and with a resolution of 22 bits (71.5 nV per bit). An active-shield cap using 128 Ag/AgCl-sintered ring electrodes and shielded co-axial cables (5–10 electrode system placements) was comfortably adjusted to each participant’s head. All electrodes were referred to the right earlobe. In addition, three electrooculograms (for horizontal and vertical EOG signals) and two electromyograms for detecting any activity related to real movement (anterior and posterior deltoids) were recorded.

**FIGURE 1:**
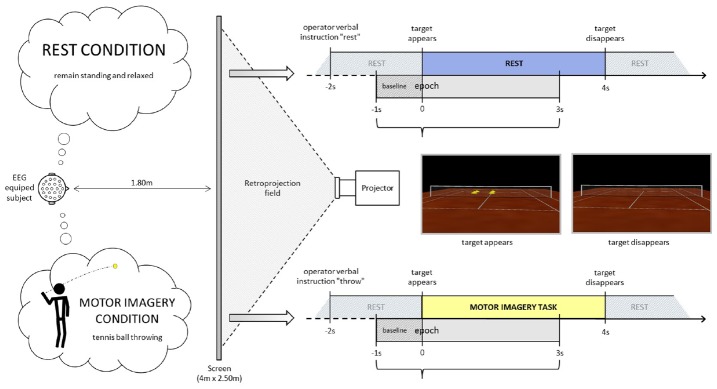
**Experimental settings.** The participant is equipped with an EEG-cap and stands up in front of a projector screen where a tennis court is displayed. He/she is asked either to remain relaxed and standing at rest or to imagine throwing a tennis ball with his/her upper limb aiming at the target (yellow cones) in the opposite court.

Participants stood erect with face and feet directed forward, arms at their sides, and the palms of their hands facing inward in a resting posture. They were positioned in front of a screen at a distance of 1.80 m from the center, where an empty tennis court was projected with a right orientation (3°), so that the participant perceived him/herself to be behind the right baseline of the court (Figure 1). The participants remained standing, at rest. At least 2 s after the operator indicated “throw” or “rest,” an identical visual target appeared in the scenario (four vertical yellow cones), in the opposite left service court (with respect to the participant), for 4 s. Following the “throw” indication, the participants were asked to imagine holding a tennis ball in their dominant hand (right in the present population) and throwing it as soon as the target appeared, aiming at the target (four vertical yellow cones), in the opposite left service court. This task does not require any particular motor expertise. Participants were also instructed to stay focused on the imaginary ball they had just thrown until target disappeared. Following the “rest” indication, the participants were asked to remain relaxed and standing at rest when the visual target was presented in the virtual scenario. The visual information presented (four vertical yellow cones as visual target) was identical in both conditions (Figure 1, time-lime schemes). There were 4 or 5 series of 40 trials (20 per condition) randomly presented in a series. Breaks were planned between series. Participants did not mention feelings of tiredness during the recordings. If the operator observed noisier than usual online raw EEG signals, the participant was kindly asked to make a break and/or to stop the experiment. Thus six participants performed four series and five participants performed five series.

### EEG Analysis

Off-line, data treatment and statistics were performed by means of EEGLAB software (Delorme and Makeig, 2004). Initially, a 200 Hz low pass filter, a 512 Hz resampling and a 0.1 Hz high pass filter were applied. When necessary a 47.5–52.5 Hz notch was also applied. Then any artifactual portions of the EEG data were rejected by visual inspection. Synchronous or partially synchronous artifactual activity (mostly blinks) was detected and rejected by independent component analysis (ICA) on continuous data. For the time domain analysis, ERPs were calculated by averaging base line corrected epochs extracted from –1 to 3 s of the target apparition event (when the four yellow cones appeared on the screen scenario). After artifacts rejection, a total of 1788 epochs remained from the initial 1960 epochs (<10% rejected). The duration of each epoch represents the average duration of mental movement measured during preliminary trials (2,4 s ± 0.5), where participants indicated the end of their mental movements by means of brief vocal sounds (“top”).

For the time-frequency analysis of EEG oscillations, we first calculated the baseline-normalized spectrogram or ERSP (Makeig, 1993; Pfurtscheller and Neuper, 1994). ERSP measures variations in the power spectrum of ongoing rhythms at specific periods of time and frequency ranges. In ERSP measurements, ERD (event related desynchronization) indicates a power spectrum reduction while ERS (event related synchronization) indicates a power spectrum increase. ERD/ERS are interpreted as reflecting brain reactivity. ERSP variations are related to the specific aspect of information processing that is time-locked but not necessarily phase-locked to stimulus. Thus we also calculated the ITC (Tallon-Baudry et al., 1996) which measures consistency across trials of the EEG spectral phase at each frequency and latency window of ongoing rhythms. We used wavelet transform for complex spectro-temporal representation with Hanning-windowed sinusoidal wavelets at 0.1 cycles (lowest) to 25 cycles (highest). ERSP and ITC templates were calculated with 500 time points (–442.1 to 2440.1 ms), using a window size of 571 samples (1115.23 ms) at 100 linear spaced frequencies from 0.1 to 50 Hz. For the significance level of ERSP and ITC, a bootstrap resampling (*p* < 0.05) was used as a surrogate method.

The significance between the two experimental conditions (“throw” and “rest”) in time (ERP) and in time-frequency domain analysis (ERSP and ITC) was calculated with parametric analysis (*p* < 0.05). For the ERPs, two-tailed paired *t*-test at each latency point on the ERPs from each subject is calculated. For ERSP and ITC, *p*-values are computed at every time/frequency point. The significant frequency-time zones reported in the results follow the statistical graphical templates provided by EEGLAB. We employed the Holm’s method for the correction of multiple comparisons since we are performing 128 simultaneous *t*-tests (one for each EEG channel; Holm, 1979; McFarland et al., 2000; de Lange et al., 2008). The latter allowed the representation of ERP, ERSP, and ITC scalp topographies.

## Results

### Time Domain Analysis

**FIGURE 2:**
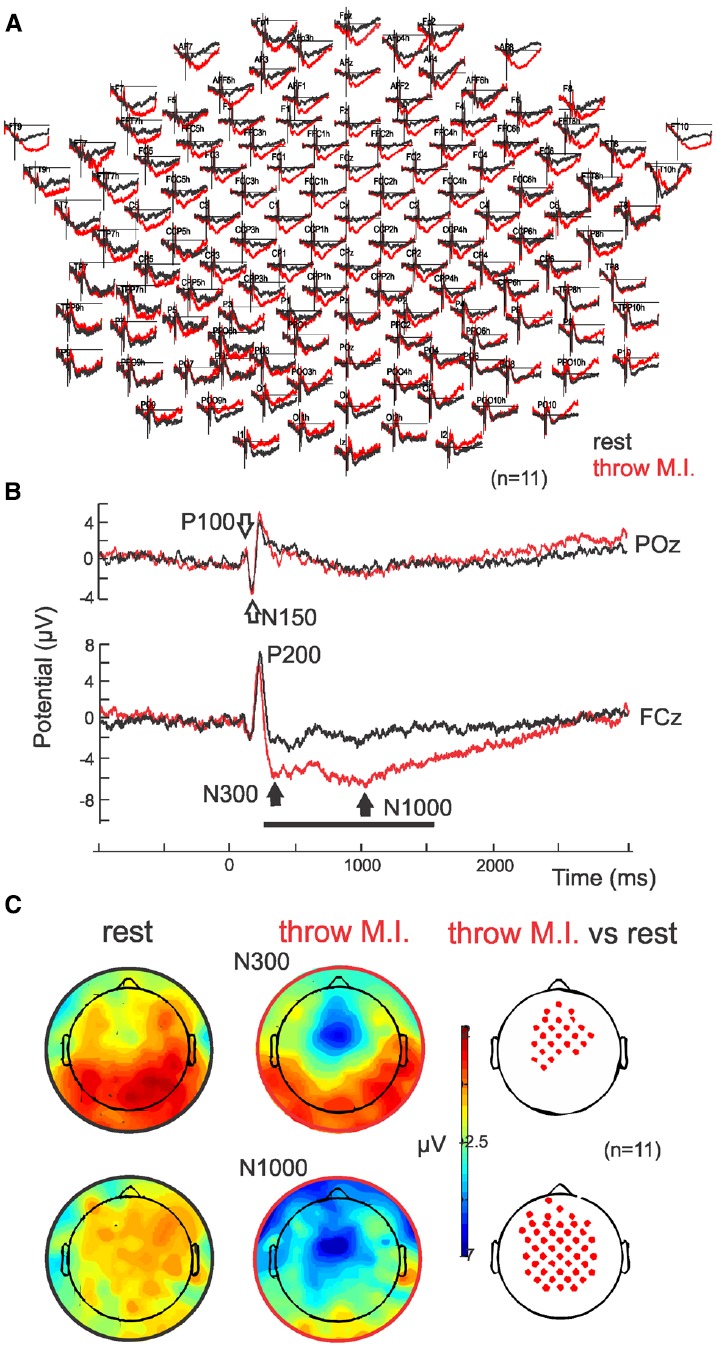
**ERPs. (A)** Grand average (*n* = 11) in full scalp array for the rest (black traces) and for the motor imagery of throwing (red traces). **(B)** ERP in POz: the classical visual P100–N150 complex is indicated with open arrows. ERP in representative electrode FCz: Note the negative wave characterized by a N300 and N1000 in the motor imagery of throwing condition. **(C)** Scalp potential topography of the N300 and N1000 components in both rest (left) and motor imagery of throwing (middle) conditions and their statistical differences (right; *p* > 0.05). Note that there is no special right or left laterality.

Parietal and occipital ERPs of both conditions (“throw” and “rest”) showed similar visual P100-N150 complex elicited by the target’s appearance on the screen with no significant differences (*p* = 0.45 and *p* = 0.50, respectively; P100 of 113.3 ± 5.6 ms and 1.4 ± 1.2 μV, N150 of 152.1 ± 8.9 ms and 3.6 ± 1.7 μV in the “throw” condition versus P100 of 111.3 ± 8.2 ms and 0.85 ± 0.9 μV, N150 of 152.3 ± 10.2 ms and 3.5 ± 1.2 μV in the “rest” condition in POz; Figures 2A,B, empty arrows). Analogous to the parieto-occipital visual complex, a positive P200 was observed in the fronto-central areas, and presented no significant difference (*p* = 0.06) between conditions (with latencies of 224.6 ± 9.1 ms and 209.0 ± 9.0 ms and amplitudes of 5.6 ± 3.9 and 7.1 ± 2.9 μV in FCz for “throw MI” and “rest” conditions, respectively). Interestingly, ERPs showed a sustained stronger fronto-central negativity wave in the “throw MI” condition with respect to the “rest” condition. This is illustrated in the grand average ERP for the whole EEG electrodes montage (Figure 2A) and for one representative electrode, FCz (Figure 2B where the black horizontal bar indicates the duration of the amplitude significant difference for *p* < 0.05). Such negativity was characterized by two minima at around 300 ms (N300) and around 1000 ms (N1000; Figure 2B, black arrows; latencies of 320.3 ± 39.6 ms and 1021.0 ± 60.7 ms in FCz) with significant (*p* > 0.05) stronger amplitudes than the “rest” condition (–6.1 ± 3.3 μV versus –2.1 ± 2.8 μV and –6.8 ± 3.9 μV versus 2.5 ± 4.0 μV for N300 and N1000, respectively with *p* = 0.001 and *p* = 0.002, respectively; Figures 2B,C). The N300 component showed a more restricted central localisation than the N1000 component, which expanded to the frontal and postcentral areas as illustrated in the topographical potential distribution maps (Figure 2C). N300 and N1000 components did not show either left or right laterality (Figure 2C).

### Time-Frequency Analysis

**FIGURE 3:**
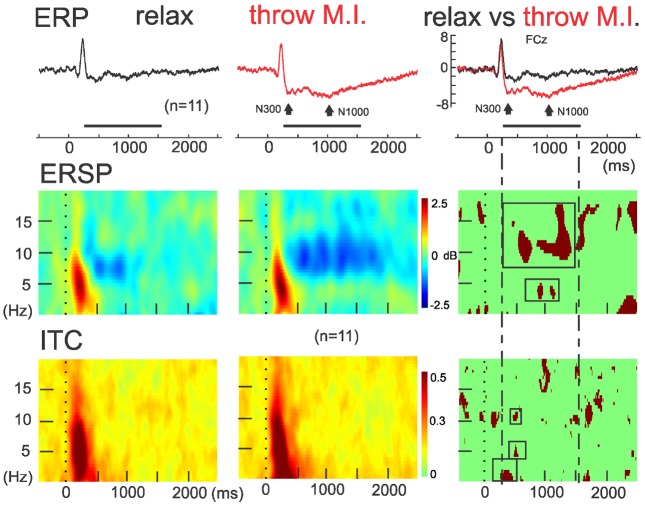
**ERSP and ITC time-frequency measurements in FCz.** Concomitants to ERPs (up), grand averaging of ERSP (middle) and ITC (down) for the rest (left) and motor imagery of throwing (middle) condition and theirs statistical differences (on the right column). Note the red significant ERSP and ITC clusters enclosed in the intermittent lines during the significant negative ERP wave in FCz.

**FIGURE 4:**
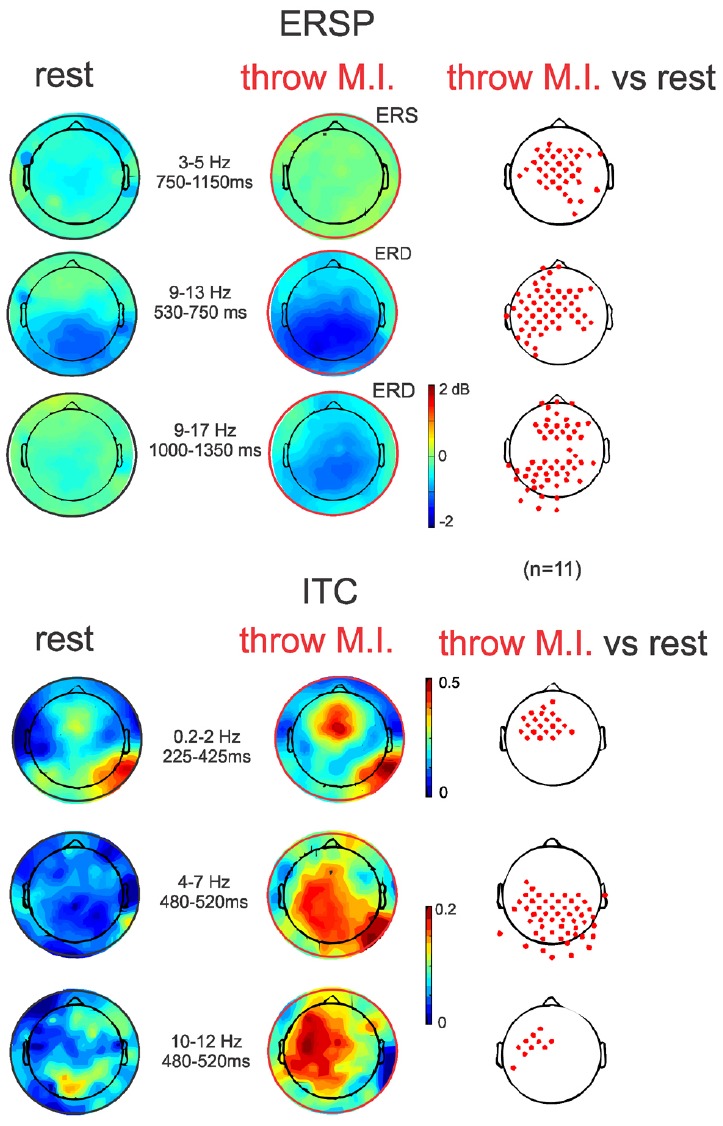
**ERSP and ITC time-frequency measurements in full scalp array topography (***n*** = 11).** ERSPs (up) and ITCs (down) topographies during the ERSP and ITC clusters of significant differences between both experimental conditions indicated in Figure 3 (red clusters in Figure 3, right column). Topographical significant differences are plotted on the right column.

The ERSP average template of FCz (Figure 3, second row) showed that both conditions presented comparable clusters of power spectrum increase (event related synchrony, ERS) centered at 5 Hz (ranging from about 0.2–10 Hz) during the first 500 ms underlying the visual processing related P200. In “rest” condition, an alpha band of power decrease (ERD), centered at 8 Hz, was observed from 300 to 1250 ms. Interestingly, during the “throw MI” condition the alpha band of ERD was centered at a higher frequency of 10 Hz (ranging from about 7 to 14 Hz) and presented a longer duration underlying the entirety of the significant negative ERP wave. In addition, the ERSP template in the “throw MI” condition showed a slight but persistent power increase band in the 3–5 Hz frequency range. The statistical template of significant differences focused on the negative ERP wave duration (including the N300 and N1000 components). ERSP significant effects were encountered below 20 Hz. A stronger ERD was found in the high-alpha/low-beta frequency band, and a stronger ERS in the theta band during the “throw MI” with respect to the “rest” condition. The clusters of significant high alpha- low beta ERD extended from 530 to 750 ms in the 9–13 Hz range and from 1000 to 1350 ms in the 9–17 Hz range. The first ERD significant cluster had left predominance from the fronto-central to the parietal scalp areas (Figure 4, ERSP, second row). The second ERD significant cluster was localized in the fronto-precentral and parieto-occipital scalp areas (Figure 4, ERSP, third row). The clusters of significant theta ERS extended from 750 to 900 ms and 1000–1150 ms in the 3–5 Hz range with a frontal and central scalp location (Figure 4, ERSP, first row).

The ITC average templates of FCz (Figure 3, third row) showed a cluster of phase synchrony centered at 5 Hz (ranging from 0.2 to 15 Hz) during the first 500 ms in the “rest” condition and wider in the “throw MI” condition. A statistical template of significant ITC differences between conditions during the negative ERP wave duration (including the N300 and N1000 components) revealed that such clusters were stronger from 225 to 425 ms at frequencies of 0.2–2 Hz, and from 480 to 520 ms at the 4–7 Hz and 10–12 Hz frequencies for the “throw MI” condition. The topography of the delta ITC cluster showed a central location with slight but significant left predominance (Figure 4, ITC, first row), that of the theta cluster had a post-central parieto-occipital location (Figure 4, ITC, second row) and that of the alpha-mu cluster had a left precentral location (Figure 4, ITC, third row). Other small, disseminated clusters appeared in the statistical template of the ITC, however, as they corresponded to the comparison of non-significant values of ITC (<0.2) in the conditions, they were discarded.

## Discussion

The present study combined both the time (ERP) and time-frequency domain measurements (power spectrum and phase synchrony variations respectively assessed by ERSP and ITC) for the characterization of the mental simulation of this simple everyday goal-directed movement of throwing a tennis ball with the dominant upper limb (the right hand in this population). When compared to the rest condition with the same visual inputs, a consistent negative fronto-central ERP wave was found. This wave was characterized by a central N300 component and an N1000 component with a wider central spread. While the ERP topographies showed no right or left dominance, oscillatory analysis showed that: (1) the N300 component was accompanied by a significant phase consistency, or ITC, of the delta band in the contralateral central scalp areas; (2) the N1000 component was accompanied by a significant low beta power decrease, or ERD, localized in fronto-precentral and parieto-occipital scalp areas and also by a significant theta ERS spreading fronto-centrally; (3) the transition of the N300 to the N1000 was accompanied by a high alpha- low beta ERD with contralateral left predominance from fronto and central to parietal scalp areas, as well as by significant phase consistency of the postcentral theta and contralateral left central alpha.

The present findings corroborate previous studies of oscillatory brain dynamics that indicated the presence of alpha-mu and beta ERD over sensorimotor regions (McFarland et al., 2000; de Lange et al., 2008) throughout the whole period of simulation being hand specific from the onset of ERD at 500 ms (Osuagwu and Vuckovic, 2014). A functional dissociation of the alpha (8–12 Hz) and beta (15–25 Hz) rhythms has been demonstrated in mental simulations of goal-directed hand actions with different levels of task demand (Brinkman et al., 2014). It was proposed that the alpha ERS in the ipsilateral sensorimotor cortex increases with higher task demands, supporting the inhibition of cortical regions irrelevant for the task. Beta ERD in the contralateral sensorimotor cortex was related to the inhibition reduction of neuronal populations involved in the computation of movement parameters in order to converge into a state suitable to action (Brinkman et al., 2014). We found an alpha-mu ERD in the central scalp areas contralateral to movement at around 530–750 ms, which may similarly be related to the activation of the specific sensorimotor areas involved in the program of motor settings. The subsequent theta ERS fronto-central spreading may be related to the activation of neural networks involved in the allocation of attention to target stimuli (Missonnier et al., 2006). Later (at around 1000–1350 ms), we found a high alpha- low beta ERD localized in the precentral-frontal areas, which may be related to motor planning, and a high alpha- low beta ERD in the parieto-occipital scalp areas (Osuagwu and Vuckovic, 2014), which may be associated to mechanisms of additional proprioceptive information (Cebolla et al., 2014) or to a visual component corresponding to the mental imagery of the ball flight.

Complementing ERSP, ITC analysis showed here a slight but significant contralateral-central phase synchrony in the delta rhythms accompanying the central N300. During the transition from N300 to N1000, a post-central and parieto-theta and a contralateral alpha (mu) rhythm occurred. The latter corroborates the study of Llanos et al. (2013), where it was shown that MI linked to a visual stimulus modified phase organization of mu rhythms, at the same time that progressive, persistent mu ERD occurred. These phase consistency changes were associated with the non-motor components of the action, such as the estimation of the probability of reaching the motor goal.

Coupling any type of sensory input to MI may interfere with the self-paced imagery process. In the present study, the visual inputs were the same during the rest and MI tasks, which produced similar theta ERS accompanying the visual components. In addition, the stability of the visual input in both situations was clearly identified by the absence of any modification of the ERP trace in the occipital region, not only during the early phase (P100 and N150) but also throughout the duration of the task. This corroborated a previous study showing that VEP failed as a predictor of successful MI task completion (Geronimo et al., 2012). Unchanged visual ERP components suggest a non-specific activation of the primary visual cortex but extrastriate involvement might be expected as the participants reported to “see” the imaginary movement in “their heads.” Intracerebral electrical stimulation of the human right visual cortex evoked visual hallucinations (Jonas et al., 2014). However, MRI measurements in Parkinson’s disease patients with diurnal visual hallucinations but without cognitive dysfunction have shown reduced gray matter substance in the left insula and the left trigonal frontal gyrus, without any special anomaly in the visual cortex which supports the concept of top-down visual processing in the genesis of visual hallucinations (Gama et al., 2014). Following this suggestion, we hypothesize that the visual representation of movement that participants may form in their minds might underlie a top-down process from fronto-central areas reflected by the amplitude changes observed in the fronto-central ERPs and by the significant phase synchrony in contralateral fronto-central delta and contralateral central mu to parietal theta presented here. Phase synchrony is considered to be the mechanism leading the dynamic integration of distributed neuronal assemblies in brain function (Friston, 1997; Tononi and Edelman, 1998); as a response to sensory cognitive event, oscillating neuronal groups would adapt their firing to a precise phase locking over a limited period of time. Phase synchrony measurements are currently used both for the study of local synchrony that reflects local integration processes, and for long distance synchrony between cortical regions reflecting large scale integration processes (Basar et al., 1999; Lachaux et al., 1999). Such top-down process, suggested by the topographical phase locking, could be analyzed in further studies by studying brain connectivity with coherence analysis between EEG channels (Nolte et al., 2004; Cheron et al., 2014) and by revealing the brain sources that account for such dynamics by using inverse methods of source reconstruction of the significant ITC values (Cebolla et al., 2011).

The similarity of cortical mechanisms between actual movement and MI have been largely supported involving the primary motor cortex, premotor cortex, supplementary motor cortex, cingulate, and parietal cortex (Beisteiner et al., 1995; Decety, 1996; Grafton et al., 1996; Porro et al., 1996; Sirigu et al., 1996; Deiber et al., 1998; Lotze et al., 1999; Gerardin et al., 2000; Naito et al., 2002; Hanakawa et al., 2003; Dechent et al., 2004; Meister et al., 2004; Stinear et al., 2006). There is a neural specificity for the different imagery perspectives and modalities (Guillot et al., 2008; Jiang et al., 2015). In this way, the dorsal stream activation of the parietal cortex supports internal visual imagery, the bilateral ventrolateral occipito-temporal cortex activation of the ventral stream supports external visual imagery and, finally, the cerebellum and basal ganglia support kinaesthetic imagery (Jiang et al., 2015).

The N300 component in VEPs has been previously described during the initial categorization of an object preceding its specific identification (Hamm et al., 2002), evidencing the influence of scene context on the recognition of ambiguous and unambiguous objects (Dyck and Brodeur, 2015) and indexing the rapid matching of a visual input to stored semantic knowledge (Schendan and Kutas, 2007; Sitnikova et al., 2008). Interestingly, an N300 component of similar central topography, as reported above, has been characterized in paradigms associating an action to a stimulus (Falkenstein et al., 1999; Ocklenburg et al., 2011; Debruille et al., 2012). Concretely, during a Go/Nogo-task where participants have to respond to one stimulus or “Go-stimuli” and to refrain from responding to the others or “Nogo-stimuli,” the N300 response corresponds to the Nogo response (Pfefferbaum et al., 1985; Falkenstein et al., 1999). Such N300 or Nogo-N2 reflects a premotor inhibition in the frontal cortex that is modality specific, with visual stimuli evoking a stronger response than auditory stimuli (Pfefferbaum et al., 1985; Jodo and Kayama, 1992; Eimer, 1993; Kopp et al., 1996). We cannot directly associate the premotor inhibition produced while refraining from an action expressed by the Nogo-N2 response to the “immobility” status during a MI task. Indeed, participants were not asked to refrain from a movement but to imagine performing it without really performing it. Moreover, the N300 component described in the present study was not overlapped by a significant ERD decrease which could reflect an inhibition of brain processes by a significant precentral delta ITC which may reflect an integration process of the premotor areas.

The choice of the control condition as a reference or baseline remains a challenge (Gusnard et al., 2001). In general, when studying the neural substrates and processes of movement and MI, both conditions are compared to each other. In a Go/Nogo-task, the go-trials are compared to the Nogo-trials. Here we used a rest condition where participants were asked to remain relaxed and at rest while standing, as we think this is a realistic control condition for the MI task. It is well accepted that there is no a simple baseline but a resting state of brain function involving a specific set of mental operations (Gusnard et al., 2001). It is a complex situation involving the dynamic interplay between conscious and unconscious processes (Cheron et al., 2009). Interestingly, it has been demonstrated that natural visual scenes induced similar brain activation patterns in human individuals (Hasson and Malach, 2006) which makes the ecological virtual reality technique especially attractive for the study of the brain dynamics of realistic tasks (Cheron et al., 2009).

In this study we found that a basic, non-sportive movement ideation performed in a virtual sport context produced a long lasting (∼1 s) cortical activity reorganization expressed by significant changes in both time (ERP) and time frequency domains (power spectrum and phase consistency).

## Author Contributions

GC conceived the study. AC wrote the first draft of the manuscript. MP designed the virtual reality protocol and performed the majority of the experiments. AC, MP, CC, AL, BD participated in the analysis of the data. All the authors participated in the interpretation of the data. AC, GC, and BD wrote the final manuscript. All authors read and approved the final manuscript.

### Conflict of Interest Statement

The authors declare that the research was conducted in the absence of any commercial or financial relationships that could be construed as a potential conflict of interest.
